# Antimicrobial peptides: features, applications and the potential use against covid-19

**DOI:** 10.1007/s11033-022-07572-1

**Published:** 2022-05-24

**Authors:** Dalia Mamdouh Mabrouk

**Affiliations:** Cell Biology Department, National Research Centre, 33 El Bohouth, St., P.O.12622, Dokki, Giza Egypt

**Keywords:** Antimicrobial peptides, Antibiotics, Covid-19, Therapeutic agents

## Abstract

**Background:**

Antimicrobial peptides (AMPs) are a diverse class of molecules that represent a vital part of innate immunity. AMPs are evolutionarily conserved molecules that exhibit structural and functional diversity. They provide a possible solution to the antibiotic-resistance crisis.

**Main text:**

These small cationic peptides can target bacteria, fungi, and viruses, as well as cancer cells. Their unique action mechanisms, rare antibiotic-resistant variants, broad-spectrum activity, low toxicity, and high specificity encourage pharmaceutical industries to conduct clinical trials to develop them as therapeutic drugs. The rapid development of computer-assisted strategies accelerated the identification of AMPs. The Antimicrobial Peptide Database (APD) so far contains 3324 AMPs from different sources. In addition to their applications in different fields, some AMPs demonstrated the potential to combat COVID-19, and hinder viral infectivity in diverse ways.

**Conclusions:**

This review provides a brief history of AMPs and their features, including classification, evolution, sources and mechanisms of action, biosynthesis pathway, and identification techniques. Furthermore, their different applications, challenges to clinical applications, and their potential use against COVID-19 are presented.

## Background

Antimicrobial peptides (AMPs) are a diverse class of molecules found in nature, and expressed in all organisms, including mammals [1]. They are indispensable components of the innate immunity, and have a broad spectrum of activity against bacteria, fungi, yeasts, viruses, as well as cancer cells [2]. Generally, AMPs are relatively short in length [10–100 amino acid (aa) residues], cationic (positively charged), and amphiphilic (hydrophobic and hydrophilic) [3]. AMPs are encoded in the genome as prepropeptides, where the signal peptide occurs at the *N*-terminus, and the antimicrobial peptide domain occurs at the *C*-terminus [4]. By post-translational modification, the mature (active) peptide is generated through proteolytic cleavage [5].

Misuse and long-term use of conventional antibiotics have led to the growing problem of bacterial drug resistance [6], which stimulated interest in the development of AMPs as the next generation anti-infectives. The key advantages of AMPs over traditional antibiotics are the immunomodulatory activities and bactericidal action of most AMPs [5], making them significant compounds in the development of novel therapeutics [7]. AMPs have several biological activities, such as wound healing [8], antiviral [9], anti-biofilm [1], anti-tumor activities [10]. Increasing evidence shows that AMPs can inhibit SARS-CoV-2 (COVID-19), paving the way for their prospective use as therapeutic drugs [11]. Apart from their prospective therapeutic applications, AMPs have different applications in various fields, such as the food industry [12], aquaculture [13], agriculture [14], and animal husbandry [15].

This review provides insight into the history, sources, classes, evolution, mode of action, biosynthesis pathway, prediction, and the different applications of AMPs. In addition, the research progress on AMPs against COVID-19 is discussed.

## History of antimicrobial peptides

In 1922, Alexander Fleming succeeded in identifying the first AMP named Lysozyme. However, the discovery of lysozyme was overshadowed in 1928, when Fleming discovered penicillin [16]. Florey, Chain, and Fleming brought the potential of penicillin in medical use to fruition, and shared the 1945 Nobel Prize in Medicine. In the 1940s and at the beginning of the golden age of antibiotics, the interest in the therapeutic prospective of natural AMPs was lost. In the 1960s, the interest in AMPs as host defense molecules was awakened in the 1960s due to the rise of multidrug-resistant microbial pathogens [6]. In 1981, α-helical AMPs named cecropins -from Cecropia silk moth- was characterized, followed by magainin from African clawed frog *Xenopus laevis,* in 1987 [17, 18]. In the 1990s, the field of antimicrobial peptides expanded rapidly, reporting over 300 peptides [19]. AMPs have since been broadly identified and characterized in approximately all organisms. Currently, more than 3324 AMPs have been deposited in the Antimicrobial Peptide Database [20].

## Classification of antimicrobial peptides

**Fig. 1:**
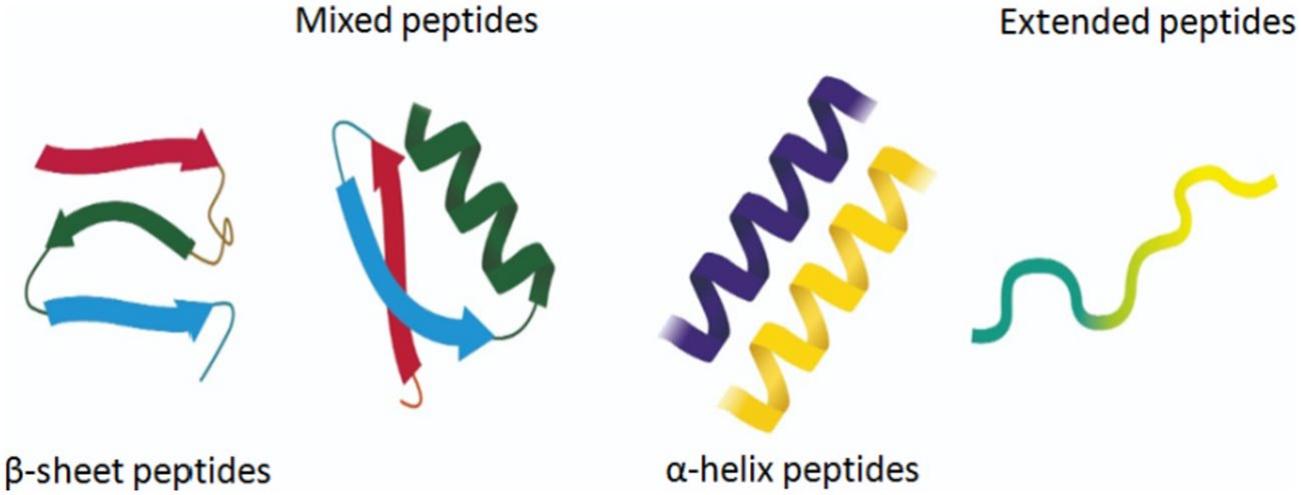
Different classes of AMPs

Based on their secondary structure, AMPs are divided into four main classes: α-helix, β-Sheet, α/β, and extended/random-coil peptides (Fig. 1). α-helix and β-Sheet are the most abundant classes in nature [3]. α-helical peptides contain plenty of helix-stabilizing residues, such as lysine, alanine, and leucine, and lack cysteine residues. The structure of α-helical peptides is lost in solution, but acquires an amphipathic helical structure in contact with a biological membrane. Frog magainins belong to this class [21]. On the other hand, β-Sheet peptides contain two to ten cysteine residues that form one to five interchain disulfide bonds. This bonding interaction allows peptides to adopt the β-sheet conformation. Unlike α-helix peptides, the structure of β-sheet peptides remains stable in aqueous solution [22]. Mammalian defensins belong to β-sheet peptides. Protegrins represent the mixed α/β class, as their secondary structure contains both α-helix and β-Sheet. The fourth class, namely extended/random-coil peptides, comprises a small part of the AMPs, and contains a high content of certain amino acids, such as arginine, tryptophan, proline, or histidine [19]. They also have greatly variable secondary structures [23]. Members of this class include the arginine and proline-rich porcine, histidine-rich human histatins, and tryptophan-rich bovine indolicidin [24].

## Sources of antimicrobial peptides

The Antimicrobial Peptide Database (APD) comprises 3324 peptides from all kingdoms: archaea (5), animals (2446), bacteria (391), protists (8), plants (364), and fungi (22), besides some synthetic peptides [20].

### Bacteria

Some AMPs are produced by bacteria to maintain the control of the population, and combat other microorganisms competing for space and nutrients in their environment. AMPs produced by bacteria are called bacteriocins. Colicins and Microcins are the two classes of bacteriocins produced by Gram-negative bacteria, while lantibiotics are produced by Gram-positive bacteria [25].

### Plants

Twelve families of AMPs are produced from plants. For example, thionins are synthesized in seeds, flowers, and leaves, whereas defensins are synthesized in seeds [25].

### Insects

Insects produce a larger range of AMPs than any other taxonomic group [26]. AMPs are synthesized in the insect fat bodies, blood cells, hemolymph, and salivary glands. Cecropins, defensins, drasomycin, and thanatin are examples of insect AMPs [27].

### Marine invertebrates

Marine invertebrates lack acquired immune response; hence, AMPs are vital for their survival. Several AMPs are detected in marine invertebrates, such as discodermins, halicylindramides, aurelins, and hedistins [28].

### Amphibians

The glands of amphibian skin are rich sources of antimicrobial peptides. The skin of frogs and toads produces numerous AMPs, such as citropins, bombinins, plasticins, and magainins [29].

### Birds

For avian species, three major classes of AMPs are identified: cathelicidins, β-defensins, and liver-expressed antimicrobial peptide-2 [30].

### Fish

In fish, AMPs are defensive weapons against devastating diseases [30]. Piscidin, hepcidin, dicentracin, and NK lysine are examples of fish AMPs [31, 32].

### Mammals

Cathelicidins and defensins are the two principal AMPs families In mammalian species [33].

## Evolution and diversification of antimicrobial peptides

Hosts and pathogens live in a never-ending struggle, with pathogens constantly escaping the host’s immune response, while the immune system of the host advances its barriers against pathogens. This arms race results in the rapid evolution of immune system-related genes [34]. On the molecular level, this combat leads to the evolutionary response, represented in mutations. As a result, natural selection may act vigorously on immune-related genes such as AMPs, which subsequently show high levels of genetic diversity [35]. Over the past years, some families of AMPs from all species have showed a high and variable level of sequence diversity. Increasing evidence shows that all organisms present a particular armory of various AMP families. This variability can play a critical role in shaping the pathogenicity of microbes [35].

On the genomic level, positive and negative selection forces can figure evolution. In the positive selection, new alleles present a great strength to an individual, and exacerbate by time, resulting in the substitution of ancestral allele in the population. Contrariwise, in the negative selection, new mutations decrease the strength, and head towards disappearing from the population [36]. The most recognized AMPs diversification mechanisms are represented in gene copy number variation, gene duplication, recombination, and allelic polymorphisms. The degree to which selection forces genetic variation within AMP genes depends on several factors, such as the density, diversity, and virulence of pathogens, in addition to the action timescale of selection, effective population size, and environmental variables [37].

The different families of defensins are the best examples of evolving AMPs, since they are age-old conserved peptides that exhibit diversification patterns, indicating a common evolutionary origin [38]. It was suggested that the first of the vertebrate defensin families is the β-defensins. The phylogenetic analysis of defensins in both vertebrates and invertebrates revealed that the relationship between invertebrate defensins, Cysteine-stabilized α-helix/β-sheet motif (CSαβ), and vertebrate defensins, β-defensins, was closer than that between α- defensins and β-defensins [38]. In addition, β-defensins are present in fish, birds, and reptiles, which are phylogenetically distant groups [39]. Interestingly, CSαβ defensin peptides display high homology among plants, fungi, and invertebrates. These findings indicate that they may share a common genetic origin [40].

## Action mechanism of antimicrobial peptides

**Fig. 2:**
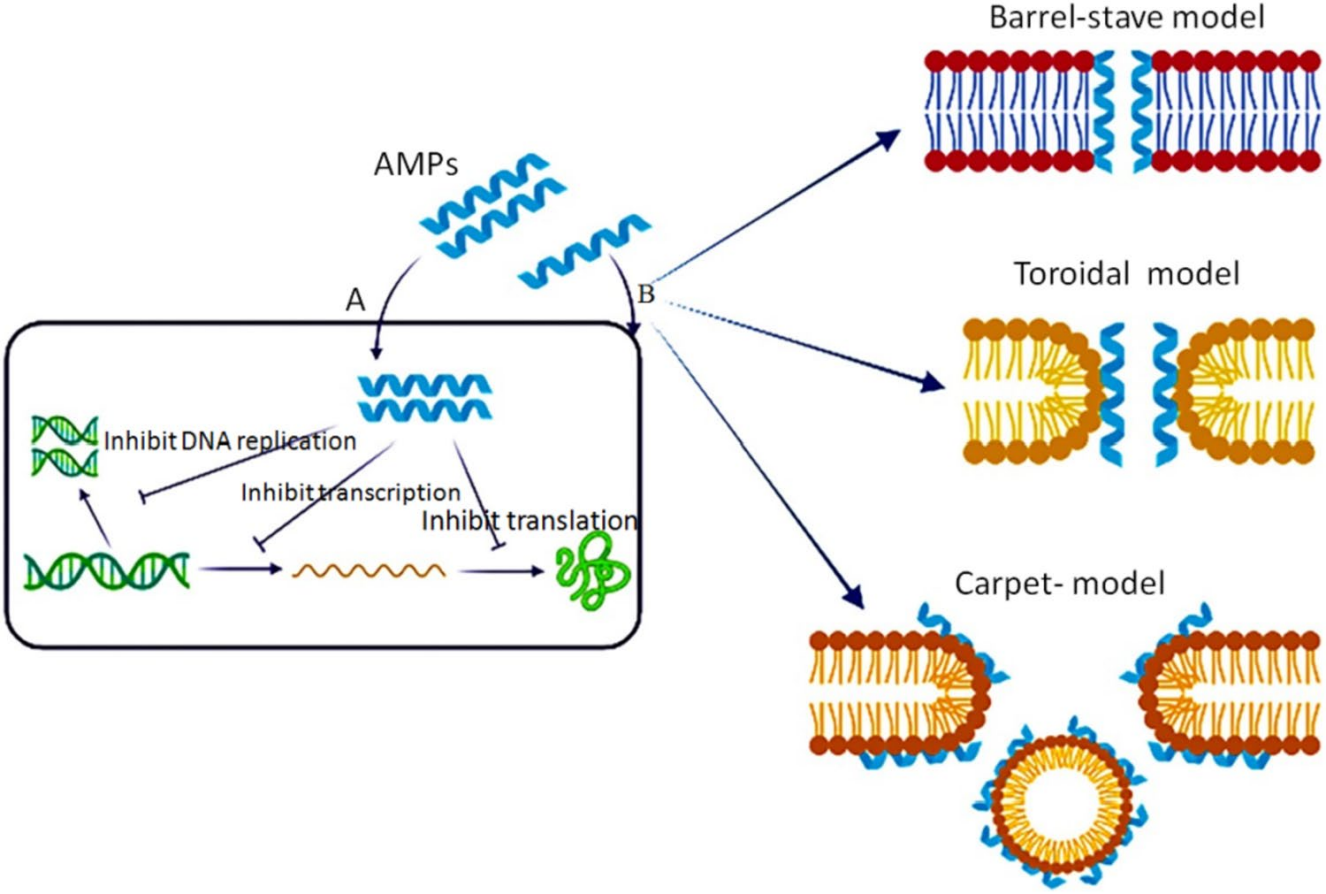
Mechanism of action of AMPs; inhibition of vital cell processes (**a**), direct pore information (**b**)

The action mechanism of AMPs depends on various physicochemical properties, such as the sequence of amino acids, structure-especially secondary structure, charge, and amphipathic property [3]. AMPs have different mechanisms by which they kill target microbes (Fig. 2) [41]. AMPs mainly interfere with the cytoplasmic membrane of the target, and may bind essential molecules in the living cells, leading to the inhibition of DNA, RNA, or protein synthesis, as well as the inhibition of certain enzymes and cell wall synthesis, in addition to the activation of autolysin, and distortion of cytoplasmic membrane (Fig. 2A) [42]. AMPs cause bacterial death through multiple and harmonizing actions, referred to as a multi-hit mechanism [7]. This strategy boosts the efficiency of AMPs to evade resistance development.

AMPs bind to bacterial membranes through three models [42] (Fig. 2B). The first is the barrel-stave model in which peptides are vertically positioned between the membrane, and attached forming an ion channel. Pardaxin and alamethicin act through this mechanism [43]. The second is the toroidal pore wormhole model in which pore formation does not originate from peptide–peptide interactions, rather from the peptide-induced curvature in the lipid bilayer; the pore is generated by both the peptide and the phospholipid head groups [44]. Many AMPs act in the toroidal model, such as protegrin-1, melittin, and magainin-2. The toroidal model shows bilayer disturbance, but remains intact in the barrel-stave model [45]. The third is the carpet model where AMPs are likely to adsorb to the membrane. Once they reach a particular concentration, they apply detergent-like effect that breaks up the membrane by forming micelles. Leucine-Leucine 37 (LL-37) and cecropin adopt the carpet model mechanism [46].

**Fig. 3:**
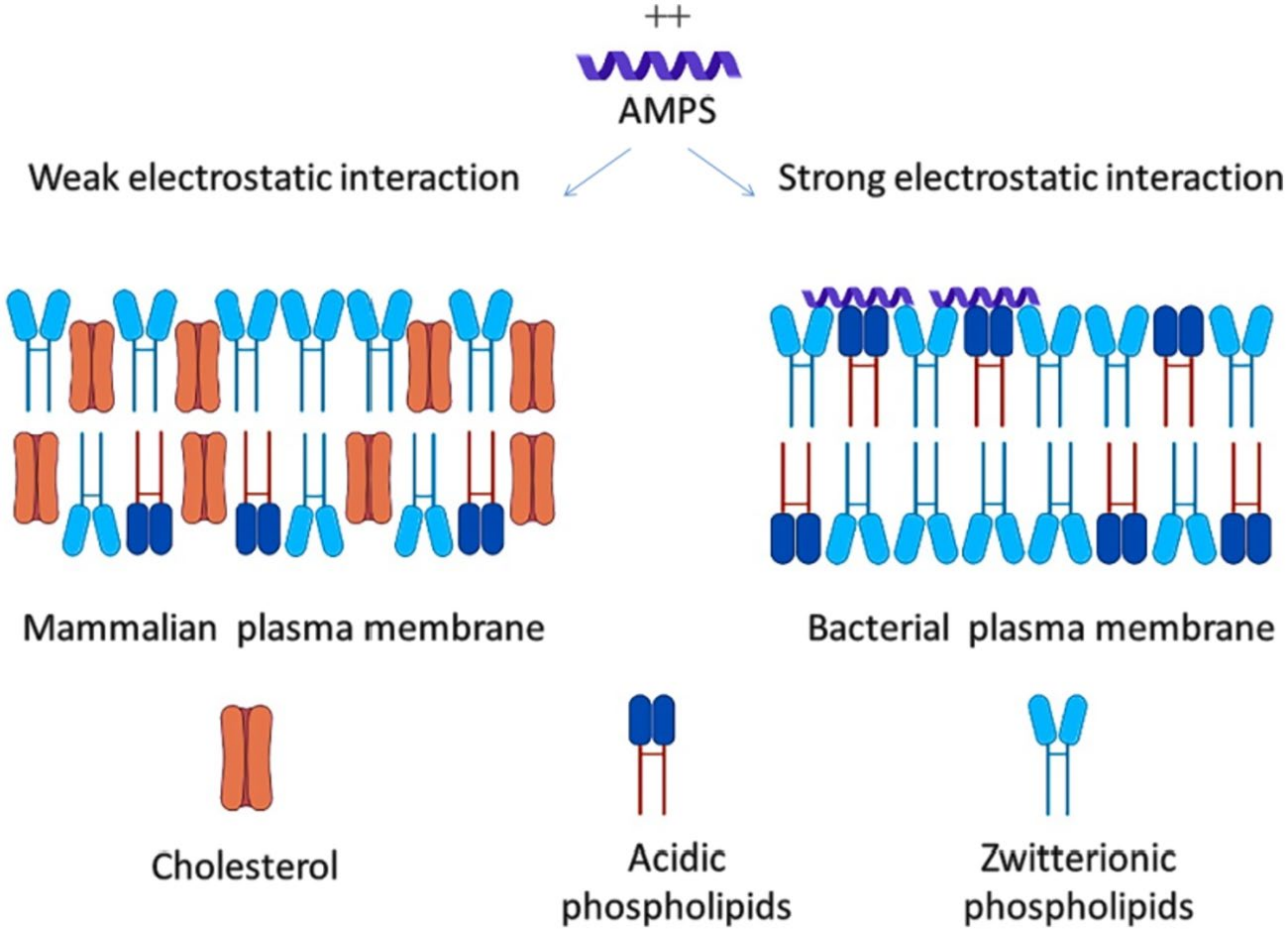
Comparison between interaction of AMP with plasma membrane of animal and bacteria

Furthermore, the cytoplasmic membrane of mammalian cells is rich in the zwitterionic phospholipids, phosphatidylethanolamine, sphingomyelin, and phosphatidylcholine, providing a membrane with a neutral net charge, while the acidic phospholipids, such as cardiolipin and phosphatidylglycerol, form the bacterial cell membranes [47]. This difference between mammalian and microbial membrane protects mammalian cells against AMPs (Fig. 3). Contrary to microbes, mammalian cell membranes have a high cholesterol content, which reduces the activity of AMPs [47]. Besides the direct bactericidal action, several AMPs have complex immunomodulatory activities, which include the induction of chemokines and cytokines, pro/anti-inflammatory activity, wound healing, angiogenesis, direct chemotaxis, apoptotic activity, and adjuvant activity [7].

## Biosynthesis pathway of antimicrobial peptides

**Fig. 4:**
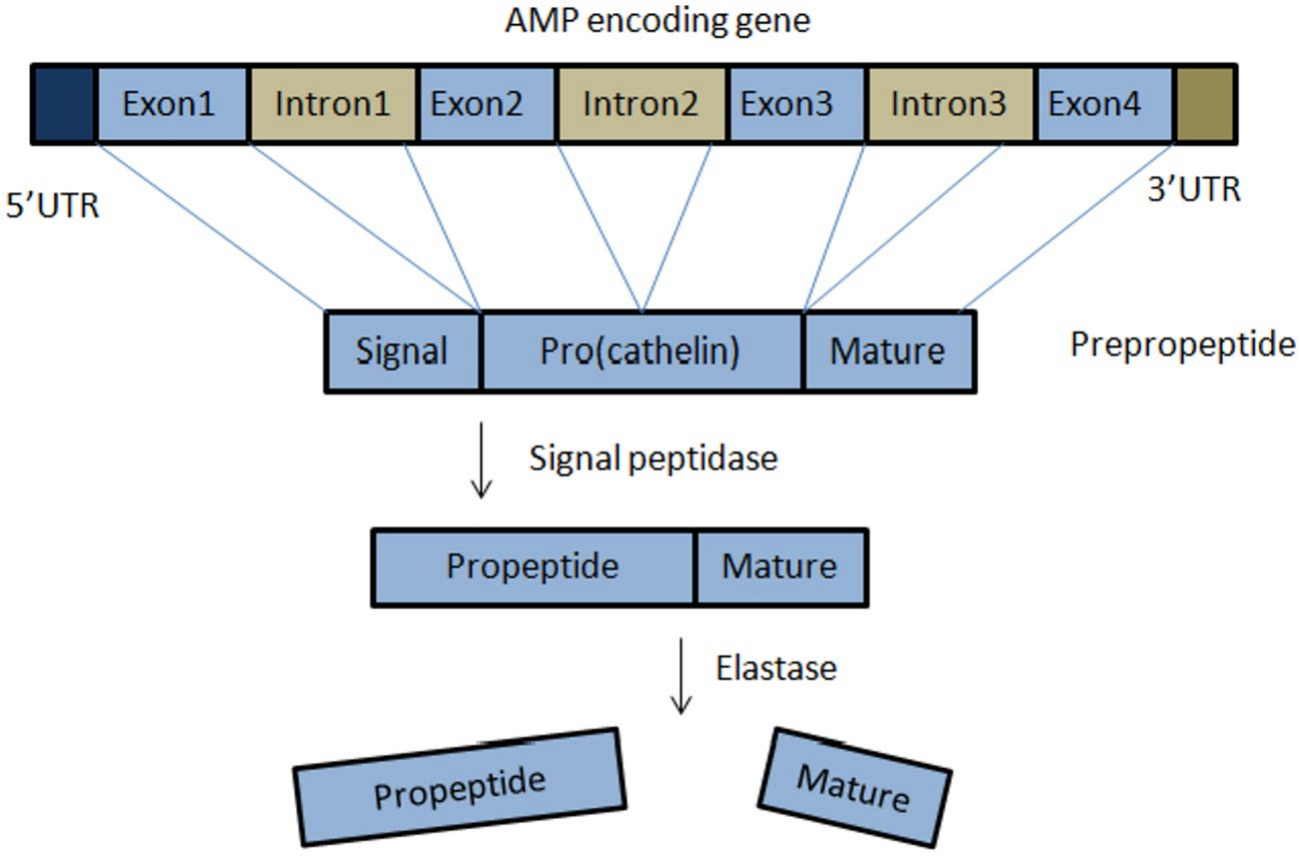
Schematic representation of precursor structure of AMPs

AMPs are produced in nature either by ribosomal translation of mRNA, or by nonribosomal peptide synthesis. Nonribosomally synthesized peptides are chiefly produced by bacteria [48], whereas ribosomally synthesized AMPs (the most common case in eukaryotes) are genetically encoded, and expressed as inactive precursors (prepropeptides) (Fig. 4). Various AMP genes such as α- and β-defensins are clustered at a single chromosomal locus, and can be co-expressed. The prepropeptide comprises a signal peptide and a pro-domain. The pro-domain keeps the mature peptide inactive until it is required. Therefore, the propeptide is anionic, while the mature peptide is generally cationic. The prodomain can have several biological functions, including intracellular trafficking, inhibition of mature peptide activity, or the correct folding of the *C*-terminus. After secretion into the extracellular space, the mature peptide is proteolytically released [48]. The pro-domain mostly occurs in the *N*-terminus, except for plants and some fish species, in which it occurs in the *C*-terminus. Usually, the signal peptide is more conserved than the mature peptide. The variability of mature peptide is a result of adaptation to challenges by pathogens. The majority of the gene-encoded AMPs undergoes post-translational modifications that are important to their function and structure [42]. Currently, there are more than 15 types of these modifications, including glycosylation, and *C*-terminal and *N*-terminal capping (amidation acetylation, and pyroglutamic acid formation), as well as disulfide-bridge formation, hydroxylation, phosphorylation, halogenation, etc. [48].

## Cathelicidins and defensins families

Cathelicidins and defensins represent the largest cationic families of AMPs. They characterize the most important part of the immune system in humans and farm animals [49]. Cathelicidins are a family of cationic AMPs widely found in mammals. This family comprises more than 30 members. While there is only one cathelicidin gene in humans and mice, there are many in other mammals [50]. LL-37 is the only member of the cathelicidin expressed in humans [42]. The genes responsible for the synthesis of cathelicidins are about 2 kb, and comprise four-exons and three introns. The first exon covers the signal peptide (part pre-) (29–30 aa). The second and third exons encode the cathelin domain (part pro-) (99–114 aa). The mature peptide (12–100 aa) is encoded by the fourth exon. Cathelicidin peptides are produced by dendritic cells, lymphocytes, NK cells macrophages, epithelial cells, neutrophils, and keratinocytes [51]. The propeptides are stored as inactive precursors, so that when the mature peptide is required, it is cleaved by neutrophil elastase to be released. The signal peptide and cathelin domain are highly conserved among species and different peptides, whereas the mature peptide shows significant heterogeneity. In some cathelicidin peptides, the C-terminal regions are α-helical, β-hairpin, or proline/arginine-rich [51].

Nonetheless, defensins are cationic peptides with a molecular mass of 3.5–4.5 kDa, containing six cysteine residues. These residues form three disulfide bridges [48]. They are divided into three classes: α-defensins, β-defensins, and θ-defensins, according to the disulfide pairings between their six conserved cysteine residues. Various AMP genes, such as α- and β-defensins, are clustered at a single chromosomal locus, and can be co-expressed. α- and β-defensins occur in the granules of neutrophils, macrophages, NK cells, epithelial tissues, skin, and in many body fluids. The triple-stranded antiparallel β-sheet structure of α-and β-defensins is stabilized by disulfide and hydrogen bonds [42]. θ-defensins are novel AMPs with a circular structure. They have been isolated from rhesus monkey neutrophils [52]. The majority of genes encoding mammalian defensins consists of only two exons; the first exon encodes the signal peptide, and the second encodes the propeptide and mature peptide [53]. The pro-peptides of α-defensins are larger than those of β-defensins. Human neutrophil defensin is processed by signal peptidase at position 19, in order to release a propeptide that is subjected to further processing by a proteolytic enzyme, trypsin in human, and metalloproteinase-7 in mouse, to release the mature peptide [42].

## Identification and prediction of antimicrobial peptides

The prediction and design of effective AMPs have significant roles in meeting the demand for novel efficient antimicrobial therapies. In the past, tissue homogenization was the first step in the identification of novel AMPs, followed by peptide extraction. Chromatographic techniques were used to isolate the crude peptide. Animals were sometimes exposed to bacterial infection or electric shocks to stimulate AMP production [48]. Afterwards, assay-guided fractionation was used to isolate potential AMPs, and the sequence was determined using special techniques, including mass spectrometry and Edman degradation. Despite its success, this approach has disadvantages, as it is time-consuming and produces low yields [48].

The rapid development and cost reductions of the next-generation sequencing technologies (NGS), in combination with efficient solid-phase synthesis techniques, enabled functional testing with no need for polypeptide isolation, nor the search for precious sequence data buried in the genome [48]. A huge amount of data, including protein, RNA, and DNA, have been generated using NGS techniques, by which peptides with antimicrobial activity could be found. Identifying AMPs is only possible through the development of computer-assisted strategies that can automatically estimate a great amount of data, and identify candidates to antimicrobial peptides before their biological evaluation in the wet lab [54]. In recent years, many computational methods have been developed to accelerate the process of antimicrobial-drug prediction and design, by providing a rational basis for candidate selection [55]. Computational research has focused on the recognition of AMPs as a means for determining which features relate to activity. Machine learning algorithms (MLA) represent the main method for training guide sequence-based classifiers to discriminate AMPs from non-AMPs [54]. Machine learning algorithms include, for example, random forest (RF), discriminant analysis (DA), support vector machine (SVM), and artificial neural network (ANN) [56]. The composition of the feature set is the critical factor for a successful prediction method, and should catch the key and accurate modules in the sequence to discriminate the real positives from negatives [55].

## Applications of antimicrobial peptides

Besides their prospective therapeutic applications, AMPs have different applications in various fields, such as the food industry, aquaculture, agriculture, and animal husbandry.

## Antimicrobial peptides as natural bio-preservative

Chemicals used in food preservation (e.g., nitrites and sulfur dioxide) may have adverse effects on the food’s nutritional value, and on human health [57]. Antimicrobial peptides have recently been used as safe food preservatives [58]. Nisin, commercially known as nisaplin™, is effective against cheese contaminating bacteria (i.e., *Staphylococcus aureus* and *Lactobacillus spp*.) [59]. It is also used for the preservation of yogurt, canned vegetables, and juice. Pediocin PA-1, marketed as Alta™ 2341, is effective against *Listeria monocytogenes* that spoils meat [12].

## Antimicrobial peptides in aquaculture

In aquaculture, the use of AMPs is a solution to troubles caused by the widespread use of antibiotics [60]. Jia et al*.* [61] observed that the daily administration of an amidated form of pleurocidin to Coho Salmon, infected with Vibrio anguillarum, reduced the accumulated mortalities. Antiviral and antifungal effects were also detected when using synthetic peptides [. Moreover, oral administration of epinecidin-1 enhanced the survival of zebrafish and grouper, infected with *Vibrio vulnificus* and *Streptococcus agalactiae* [13].

## Antimicrobial peptides in pearl farming industry

In the pearl farming industry, a bacterial infection is considered a major problem because it leads to nucleus rejections and oyster mortality. Antibiotics are used to reduce postoperative mortality, and increase pearl quality. Nevertheless, such antibiotics pose an environmental and public health issues [62]. Lately, AMPs have been applied in the pearl industry. Simon-Colin et al. [62] used tachyplesin combined with exopolysaccharides, as filming agents, to reduce oyster post-operative mortality, and increase pearl quality.

## Antimicrobial peptides in plants

Transformation with gene-encoding AMPs is currently a promising approach to develop transgenic crops resistant to pathogens, thus reducing the use of harmful pesticides in agriculture [14]. Zainal et al. [63] suggested that defensins expression of chili (*Capsicum annuum*) in tomatoes improved the resistance of tomatoes to *Fusarium sp*. Giacomelli et al*. *[64] revealed that the presence of small defensin-like sequence genes in the genome of the grapevine could inhibit the conidial germination of *Botrytis cinerea* in the fruit.

## Antimicrobial peptides as feed additives

Recently, AMPs have been used as feed additives. Liu et al. [65] recommended AMPs as feed additives for goats in commercial farms, due to their beneficial effects on growth performance, rumen morphology of juvenile goats, and ruminal fermentation function. Peng et al. [66] demonstrated that dietary supplementation with crude recombinant porcine β-defensin 2 had useful effects on the growth and intestinal morphology of weaned piglets, reducing a number of potential pathogens in the caecum, and the incidence of post-weaning diarrhea. Chen et al. [67] revealed that dietary supplementation with AMP improved egg production of hens during the late laying period.

## Applications in animal husbandry

AMPs have the ability to overcome difficulties associated with conventional antibiotics, and enhance the quality of animal production [15]. Kerr et al. [68] reported that the expression of a bioactive variant of lysostaphin in the mammary glands of mice suppressed *Staphylococcus aureus*, the major infectious mastitis pathogen. Donovan et al. [69] succeeded in creating transgenic dairy cows that expressed lysostaphin in their mammary epithelium, as a step to prevent and cure of mastitis.

## Therapeutic applications of antimicrobial peptides

AMPs appear to be promising therapeutic drugs for different skin and soft tissue infections. They present a broad spectrum of antimicrobial activity, wound-healing promoting activities, such as angiogenesis, and induction of cell migration and proliferation, in addition to immune-modulatory activity [70]. For example, cathelicidin LL-37 has been used as a local treatment for leg ulcers [71]. A set of AMPs was approved by the food and drug administration (FDA) for clinical use. For example, Omiganan has been established as a local treatment for catheter infections [72]. Daptomycin (approved in 2003), Omiganan (approved in 2014), telavancin (approved in 2014), and dalbavancin (approved in 2009) are used for injection against complicated skin, and skin structure infections caused by different Gram-positive bacterial infections [8]. AMPs also have the potential to prevent sexually transmitted infections. For example, LL-37 has an antimicrobial effect against *Chlamydia trachomatis*, *Candida albicans*, and human immunodeficiency virus (HIV) [73]. AMPs could be used as novel contraceptive microbicides. For example, VRP is a small synthetic peptide that can arrest sperm motility without damaging the vaginal epithelial cells [74].

Furthermore, AMPs with anti-tumor activity, called anticancer peptides (ACPs), are new drugs that may overcome the problems associated with tumor resistance to conventional chemotherapy. In recent years, the number of natural AMPs that have antitumor activity has increased [10]. Several AMPs target tumor cells through binding the negatively charged phospholipids, i.e. phosphatidylserines (PS), localized in the outer leaflet of plasma membranes [75]. The outer leaflet of the plasma membrane of normal human cells contains neutral phospholipids, i.e. phosphatidylcholines and sphingomyelins, while the inner leaflet contains negatively charged PS. Nevertheless, this condition is inverted in cancer, owing to oxidative stress, acidity, thrombin, and inflammatory cytokines [10]. The high fluidity of cancer cells, due to the decreased levels of cholesterol, facilitates AMP-induced apoptosis [76]. Numerous ACPs have lately been discovered. For example, Cecropin B exhibits anticancer activity against lung and stomach cancer, and leukemia cells [1]. Bacteriocin was found to have cytotoxic activity against various human cancer cells [77]. Qin et al. [78] revealed that the novel modified peptide of cecropin B, CB1a showed an apoptosis activity in carcinoma cells.

Besides the direct administration of AMPs, several agents induce the expression of AMPs by the body to improve immune responses [7]*.* Vitamin D3 was shown to increase the gene expression of cathelicidin [79]. Recently, it has been suggested that the combination of AMPs with traditional antibiotics increases their efficiency [80].

## Challenges to clinical applications

Limitations on the clinical applications of AMPs are imposed by the susceptibility to proteolytic degradation, toxicity, specificity, immunogenicity concerns, rapid clearance from the kidney and liver, hemolytic activity, and delivery issues [81, 82]. For instance, whereas Indolicidin has a broad spectrum of antimicrobial activity, it exhibits hemolytic activity that limits its clinical application [3]. Furthermore, several of these cationic AMPs were reported to be more or less toxic to human cells [83]. Immunogenicity is also a serious obstacle in peptide drug development, although AMPs are small molecules with little to no immunogenicity. As a result, improving the safety of AMPs is a critical concern in drug research and development [3].

Different strategies have been explored to obtain safer AMPs as a potential drug candidate [73]. The formulation and chemical modification of the peptides are among the most commonly used strategies. Natural AMPs can be modified chemically by glycosylation [84], non-natural amino acids incorporation [85], acetylation, lipidation, and cyclization [86]. Kumar et al. [87] reported an increase in proteolysis stability of AMPs when positively-charged arginine residue was substituted with charged non-natural amino acids (e.g. L-ornithine and L-homoarginine). Wang et al. [88] utilized a peptidomimetics strategy, in which polymers with a modified backbonewere used to prevent proteolysis, and increase stability. Diverse delivery systems, such as surfactant/lipid-self assembly systems, inorganic materials, and polymers, were used to improve the half-life, stability, and toxicity of AMPs [89]. Lately, nanotechnology has been used as a convenient system to control the release and the delivery of AMPs [90].

## Potential use against COVID-19

Coronaviruses (CoVs) are a family of viruses that has the ability to infect various animals, and cause respiratory illness in humans. Severe acute respiratory syndrome coronavirus (SARS-CoV), and Middle East respiratory syndrome coronavirus (MERS-CoV) are examples of extremely pathogenic coronaviruses that emerged in humans, causing fatal respiratory illness [91]. COVID-19 is an infectious disease detected in China in 2019, and its causative agent is SARS-CoV-2. It has resulted in global deaths that exceeded 5.5 million since its detection (https://coronavirus.jhu.edu/map.html). Considering the acute crisis of COVID-19 pandemic, there is an urgent need for developing effective antiviral therapeutics for the prevention and treatment of COVID-19.

**Fig. 5:**
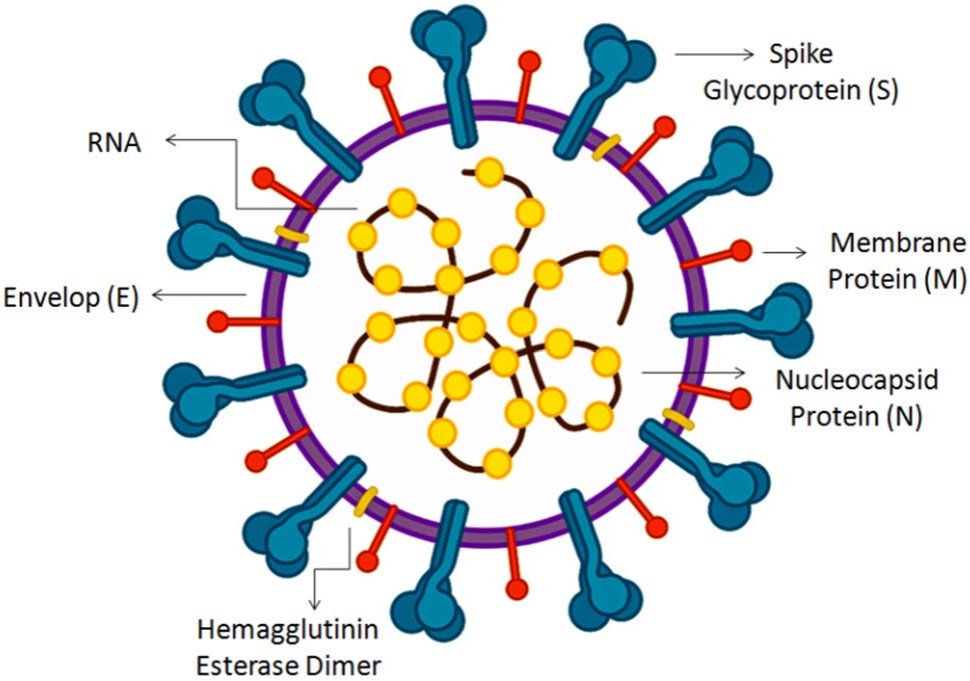
Schematic figure of coronavirus structure

CoVs are enveloped viruses with single-stranded ribonucleic acid (ssRNA). Their genome is considered the largest RNA virus (26 to 32 kb). The 3ʹ terminus of SARS-CoV-2 encodes envelope (E), spike (S) glycoproteins, nucleocapsid (N) proteins, and membrane (M) glycoproteins (Fig. 5) [92, 93]. The viral infection in humans is initiated by the binding of receptor-binding domain (RBD) of spike protein to human angiotensin-converting enzyme 2 (hACE2) receptor. It is mainly expressed on the epithelial cells of lung; thus, the main target of SARS-CoV-2 is the lung [94]. In addition to the genes that encode structural proteins, certain regions at the genome encode non-structural proteins, such as coronavirus’ main protease (3CLpro), and papain-like protease (PLpro), and the viral proteins required for replication [95]. Therefore, the targets of antiviral drugs are generally the blockage of host cell surface receptors, virolysis (direct interaction with virus), inhibition of viral replication, and the inhibition of viral fusion to host cells [96].

Despite the partial protection conferred by the present vaccines against viruses prior to exposure, antiviral drugs are the first line of defense when individuals are already infected. However, a specific antiviral with high efficiency against SARS-CoV-2 is not yet available [97]. Among the 3324 discovered or synthesized AMPs, 190 peptides have antiviral activity, and called antiviral peptides (AVPs) [9]. Since AMPs have inhibitory effects on the known proteins of the SARS-CoV-2 virus, their usage is a significantly promising solution for the treatment of COVID-19 [98].

The lectin-like human defensins-5 peptide (HD5) was reported to have high-affinity binding with ACE2, protecting host cells from viral infection [94]. Bakovic et al. [99] suggested that brilacidin (a de novo designed synthetic AMP) had a potent antiviral activity in vitro against SARS-CoV-2. Its proposed mechanisms include preventing viral binding to cells, and enhancing the integrity of the viral membrane. Using in silico molecular docking studies, it was found that several AVPs could prevent COVID-19. Fakih [100] confirmed that dermaseptin-S9 peptide could prohibit the attachment of the spike protein of SARS-CoV-2 to the surface of the ACE-2 receptor. Liscano et al. [101] reported that AMPs might be selective for viral proteins, concluding that caerin 1.6 and caerin 1.10 (Amphibian AMPs) had the ability to interact with Sgp, yet had low affinity for ACE2 proteins. More recently, Bhattacharya et al. [102] unraveled that the binding affinity of the natural food preservative peptide, Nisin, to hACE2 was higher than that of the spike protein of SARS-CoV-2. Furthermore, using molecular docking and molecular dynamics simulations, Balmeh et al. [98] revealed that glycocin F and lactococcine G were the best bio-AMPs to block RdRp, 3CL, S, and N proteins of SARS-CoV-2, with minimal side effects. It was found that the antibacterial peptide DP7 had a potential effect on inhibiting SARS-CoV-2 infection, through SARS-CoV-2 S protein pseudovirus infection of ACE2-293 T cells, SARS-CoV-2 S protein-mediated cell–cell fusion, and the inhibition of SARS-CoV-2-3CLpro enzyme activity [103]. These findings reported that AVPs could be effective in treating COVID-19 infection. Nevertheless, experimental and preclinical studies are necessary to assay their therapeutic effects.

## Conclusion

Since the discovery of lysozyme in 1922, more than 3324 AMPs have been recognized in most organisms. AMPs are evolutionarily conserved molecules. These small cationic peptides exhibit structural and functional diversity, and have the ability to target bacteria, viruses, fungi, and parasites, as well as cancer cells. The accelerating growth of antimicrobial resistance has deepened the need for the discovery of novel antimicrobial agents. Some limitations are still imposed on pharmaceutical development, due to immunogenicity, proteolytic degradation, and toxicity. Recently, different strategies have been explored, including chemical modifications, and modern techniques, such as drug delivery, in order to obtain safer AMPs as potential therapeutic agents. Increasing evidence shows that AMPs can inhibit SARS-CoV-2 (COVID-19), paving the way for their prospective use as therapeutic drugs.
